# ^99m^Tc-FAPI-04 SPECT/CT outperforms contrast-enhanced CT in detecting metastasis in postoperative patients with colorectal cancer

**DOI:** 10.3389/fmed.2024.1462870

**Published:** 2024-09-23

**Authors:** Donghua Sun, Li Ma, Yan Liu, Caili Bao, Guorong Jia, Tao Wang, Yingqiu Wang

**Affiliations:** ^1^Department of Nuclear Medicine, Yangpu Hospital, School of Medicine, Tongji University, Shanghai, China; ^2^Department of Nuclear Medicine, The First Affiliated Hospital of Naval Medical University, Shanghai, China

**Keywords:** ^99m^Tc-FAPI-04, SPECT/CT, contrast-enhanced CT, colorectal cancer, metastasis

## Abstract

**Purpose:**

To compare the performance of ^99m^Tc-FAPI-04 SPECT/CT and contrast-enhanced CT (CECT) in the detection of postoperative metastasis in patients with colorectal cancer (CRC).

**Methods:**

The postoperative patients with CRC were consecutively recruited from January 2023 to June 2023, and the enrolled patients completed ^99m^Tc-FAPI-04 SPECT/CT imaging and CECT examination within two weeks. Histopathological analysis and the follow-up results were used as the reference criteria. The location and number of metastatic sites and the detection accuracy between the two imaging methods were compared. The tumor-to-background ratio (TBR) of liver metastasis and lymph node metastasis in ^99m^Tc-FAPI-04 SPECT/CT imaging were also calculated for comparison.

**Results:**

In total, 19 postoperative CRC patients, including 15 patients with metastasis, were included in this study. In the patient-based analysis, ^99m^Tc-FAPI-04 SPECT/CT showed a significantly higher sensitivity for the detection of metastasis than CECT (93.3% vs. 80.0%, *p* = 0.038), but both techniques had the same specificity (100%, 4/4). For the lesion-based analysis, the detection rates of metastatic sites were 92.2% (47/51) and 72.5% (37/51) for ^99m^Tc-FAPI-04 SPECT/CT and CECT, respectively, and the difference between them was statistically significant. In the diagnosis of liver metastasis and lymph node metastasis, ^99m^Tc-FAPI-04 SPECT/CT both exceeded CECT. Additionally, the TBR in lymph node metastasis was higher than that in liver metastasis.

**Conclusion:**

The findings suggested that ^99m^Tc-FAPI-04 SPECT/CT could detect metastasis more effectively than CECT, especially liver and lymph node metastases, in postoperative CRC patients.

## 1 Introduction

Colorectal cancer (CRC) is the third most commonly diagnosed cancer in the world and ranks second in the number of cancer-related deaths, seriously damaging public health (1). Curative surgical resection is the main method of treating CRC patients, but approximately 30–50% of postoperative patients face the risks of relapse or metastasis. Thus, new strategies are needed for early detection and corresponding intervention to improve clinical outcomes (2). Contrast-enhanced CT (CECT) is routinely used to monitor CRC recurrence and metastasis after surgery because of its widespread application and high-resolution assessment of anatomical structures (3). Nuclear medicine imaging modalities, including single-photon emission computed tomography/X-ray computed tomography (SPECT/CT) and positron emission tomography/CT (PET/CT), can efficiently provide functional and morphological information on lesions, and they also have the advantage of whole-body scan; these reasons make them suitable for detecting tumor recurrence and metastasis. The ^18^F-fluorodeoxyglucose (^18^F-FDG) PET/CT technique is used as the standard-of-care imaging for initial staging and re-staging, treatment response, follow-up, and postoperative surveillance of CRC patients (4, 5).

Fibroblast activation protein (FAP) is overexpressed in cancer-associated fibroblasts (CAFs) in more than 90% of epithelial tumors, including CRC, but its level of expression in normal organs is low (6, 7). FAP-targeted PET/CT using ^68^Ga or ^18^F-labeled FAP inhibitors (FAPIs) is a highly promising imaging tool for the diagnosis, staging, monitoring recurrence, and metastasis of various cancers (8, 9). Recent studies have suggested that ^68^Ga-FAPI-04 PET/CT has higher sensitivity and specificity than ^18^F-FDG in detecting primary tumors and metastases in patients with CRC (10–12). However, the lack of availability and high cost of PET/CT scanning facilities greatly limits routine clinical use. Generally speaking, PET imaging system using the coincidence detection of two γ-rays with an energy of 511 keV that are produced when a positron released by the positron emitter radionuclide (e.g., ^18^F and ^68^Ga) annihilates with an electron, has higher spatial resolution and sensitivity than SPECT imaging, which employs physical collimator to reject photons [e.g., 140 keV photons from the gamma-ray emitter technetium-99m (^99m^Tc)] that are not within a small angular range (13). Although ^68^Ga-PET gives better image quality than ^99m^Tc-SPECT, the latter is cheaper and more feasible. Thus, SPECT as a widely used molecular imaging technique remains a good choice for the diagnosis and treatment follow-up of tumors. Herein, we conducted a comparative study on the performance of ^99m^Tc-labeled FAPI-04 SPECT/CT and CECT in detecting metastasis in postoperative patients with CRC.

## 2 Population and methods

### 2.1 Patient population

All patients provided written informed consent before the study. Postoperative CRC patients with suspected metastases who had not previously undergone chemotherapy or targeted therapy were enrolled from January 2023 to June 2023. For the enrolled patients, ^99m^Tc-FAPI-04 SPECT/CT imaging and CECT examination were performed for the evaluation of tumor metastasis, which were completed within 2 weeks.

### 2.2 Image acquisition

The precursor HYNIC-FAPI-04 kit containing 20 μg of HYNIC-FAPI-04, 10 mg of ethylenediamine-NcN′-diacetic acid (EDDA), 20 mg of tricine and 50 μg of SnCl_2_ was purchased from Shanghai Nice-labeling Biotechnology Co. Ltd. ^99m^Tc-NaTcO_4_ was provided by Shanghai Atom Kexing Pharmaceutical Co., Ltd., and the radiolabeling was performed according to the instruction manual (Supplementary Figure S1). Briefly, 2 mL of freshly eluted ^99m^Tc-NaTcO_4_ solution (2.22 GBq) was mixed with the precursor kit, and then was heated at 100°C for 15 min to prepare ^99m^Tc-HYNIC-FAPI-04 (abbreviated as ^99m^Tc-FAPI-04). Before each use, the labeling rate of ^99m^Tc-FAPI-04 was measured using a mini-scan radio-thin layer chromatography (TLC) scanner, in which silica gel paper strip and acetone was used as the stationary phase and mobile phase, respectively. Generally, ^99m^Tc-FAPI-04 exhibits an excellent labeling rate (over 95%, Supplementary Figure S2) and a high specific activity of >650 MBq/nmol, meaning that the radiotracer can be used directly without additional purification.

For SPECT/CT examination, the patients received an intravenous injection of 740 MBq of ^99m^Tc-FAPI-04, and then, whole-body SPECT imaging and SPECT/CT fusion images were taken using the GE Discovery (NM/CT 670) SPECT/CT system 60 min after injection. For CECT examination, the patients first underwent CT plain scanning, and then, they were injected intravenously with 80–120 mL of Iodixanol (270 mg/mL, Yangtze River Pharmaceutical Group Co., Ltd., China), followed by enhanced CT scanning at the artery phase (30 s delay) portal venous phase (65 s delay) using a Siemens SOMATOM Definition AS 64-row 128-slice 4D spiral CT scanner.

### 2.3 Image analysis

The ^99m^Tc-FAPI-04 SPECT/CT images were analyzed by two experienced nuclear medicine physicians. In SPECT/CT fusion images, the sites with local radioactivity accumulation and simultaneous positive CT scan results were diagnosed as having metastatic lesions. To determine the tumor-to-background ratio (TBR), the tumor region of interest (ROI) was delineated on the largest cross-section of the lesions in the co-registered SPECT/CT images, and the system software would give the radioactive counts per unit automatically. Similarly, ROI with the same size was drawn in muscle as the background. Lastly, the TBR was calculated by comparing the radioactive counts per unit volume of metastasis sites with that in the background. For CECT, images were analyzed by two senior abdominal radiologists using the radiological criteria. Generally, lesions were suspected based on temporal criteria (recent appearance) and morphological criteria (nodules or masses without typical benign characteristics).

### 2.4 Statistical analysis

All statistical analyses were performed using SPSS 20.0. The measurement data were expressed as the mean ± standard deviation (SD). The differences in the rates between the two methods were compared by *χ*^2^-test (or the Fisher exact test when appropriate), and *p* < 0.05 was considered to be statistically significant.

## 3 Result

### 3.1 Patient characteristics

The study included 19 postoperative patients with CRC, including 15 males and 4 females (average age: 59.53 ± 13.39 years). The primary tumors included 3 ascending colon cancers, 4 transverse colon cancers, 4 descending colon cancers, and 4 rectal cancers. The median time of the postoperative course was 18.42 months (range: 1–60 months), and the mean duration of postoperative follow-up was 95.16 days (range: 45–180 days). Metastasis was confirmed by tissue biopsy or repeated postoperative pathological, clinical, and imaging follow-up results. All participants underwent ^99m^Tc-FAPI-04 SPECT/CT imaging and CECT examination without serious adverse effect. The characteristics of the patients are summarized in Table 1.

**Table 1:** Summary of the characteristics of the patients.

Characteristic	Value
No. of patients	19
Sex	
Male	15
Female	4
Age (years)	
Mean ± SD	59.53 ± 13.39
Primary tumor	
Ascending colonic adenocarcinoma	3
Transverse colonic adenocarcinoma	4
Sigmoid colonic adenocarcinoma	4
Rectum adenocarcinoma	4
Postoperative time course (months)	
Mean (range)	18.42 (1, 60)
Postoperative follow-up time (days)	
Mean (range)	95.16 (45, 180)

SD, standard deviation.

### 3.2 Comparative assessment of the performance of metastasis detection

**Figure 1:**
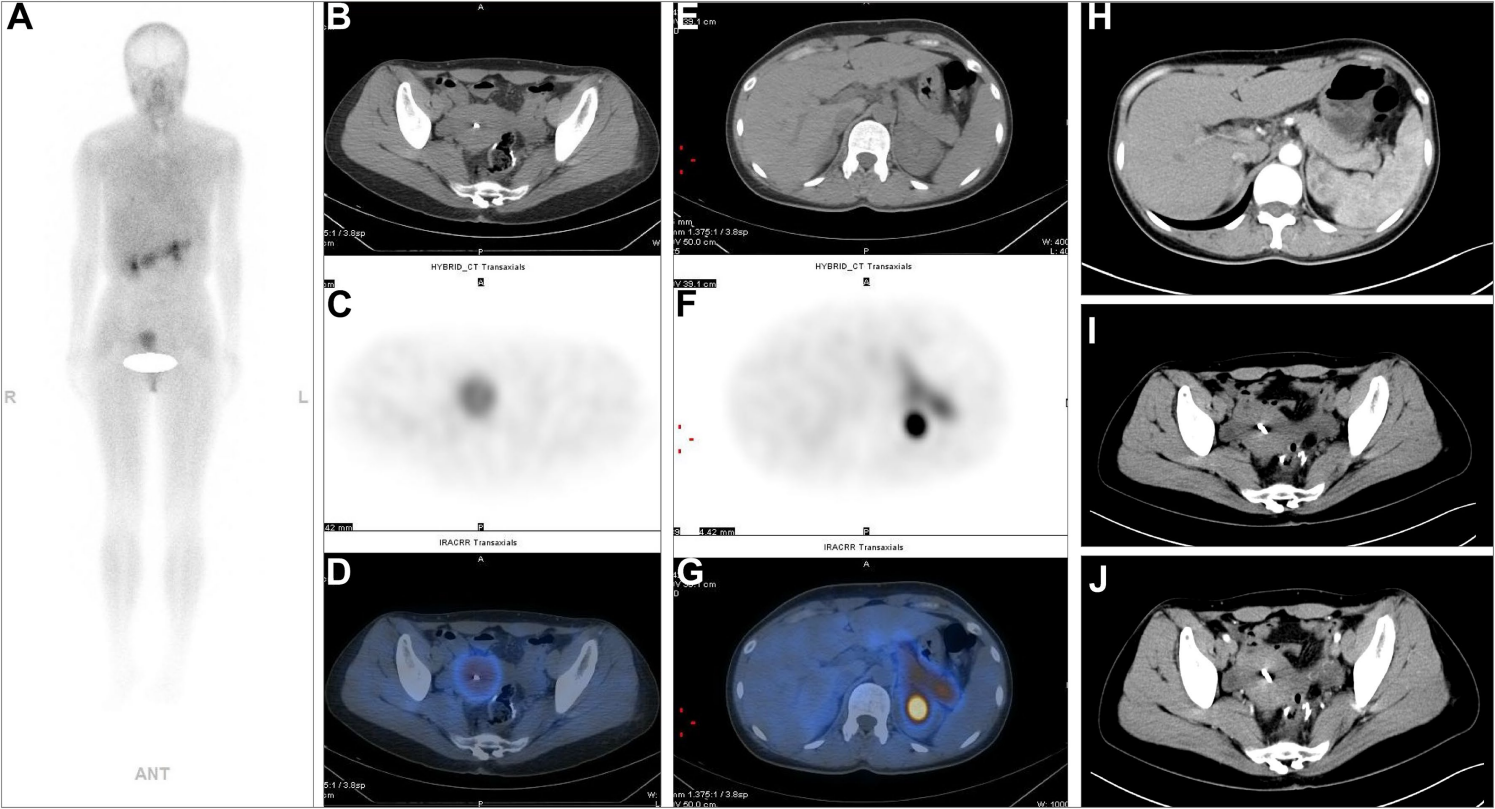
A 36-year-old postoperative female patient with sigmoid colon adenocarcinoma underwent ^99m^Tc-FAPI-04 SPECT/CT and CECT examinations. The whole-body maximum intensity projection (MIP) image **(A)**. The axial CT image **(B,E)**, axial SPECT image **(C,F)**, and the corresponding fused ^99m^Tc-FAPI-04 SPECT/CT image **(D,G)**. Metabolic foci with abnormal ^99m^Tc-FAPI-04 uptake were not detected on whole-body SPECT/CT. ^99m^Tc-FAPI-04 had high physiological uptake in the normal pancreas and uterus. Neither of the techniques showed thickening of the bowel wall at the anastomotic site, and enlarged lymph nodes were not detected in the abdominal or pelvic cavity via abdominal CECT **(H–J)**.

**Figure 2:**
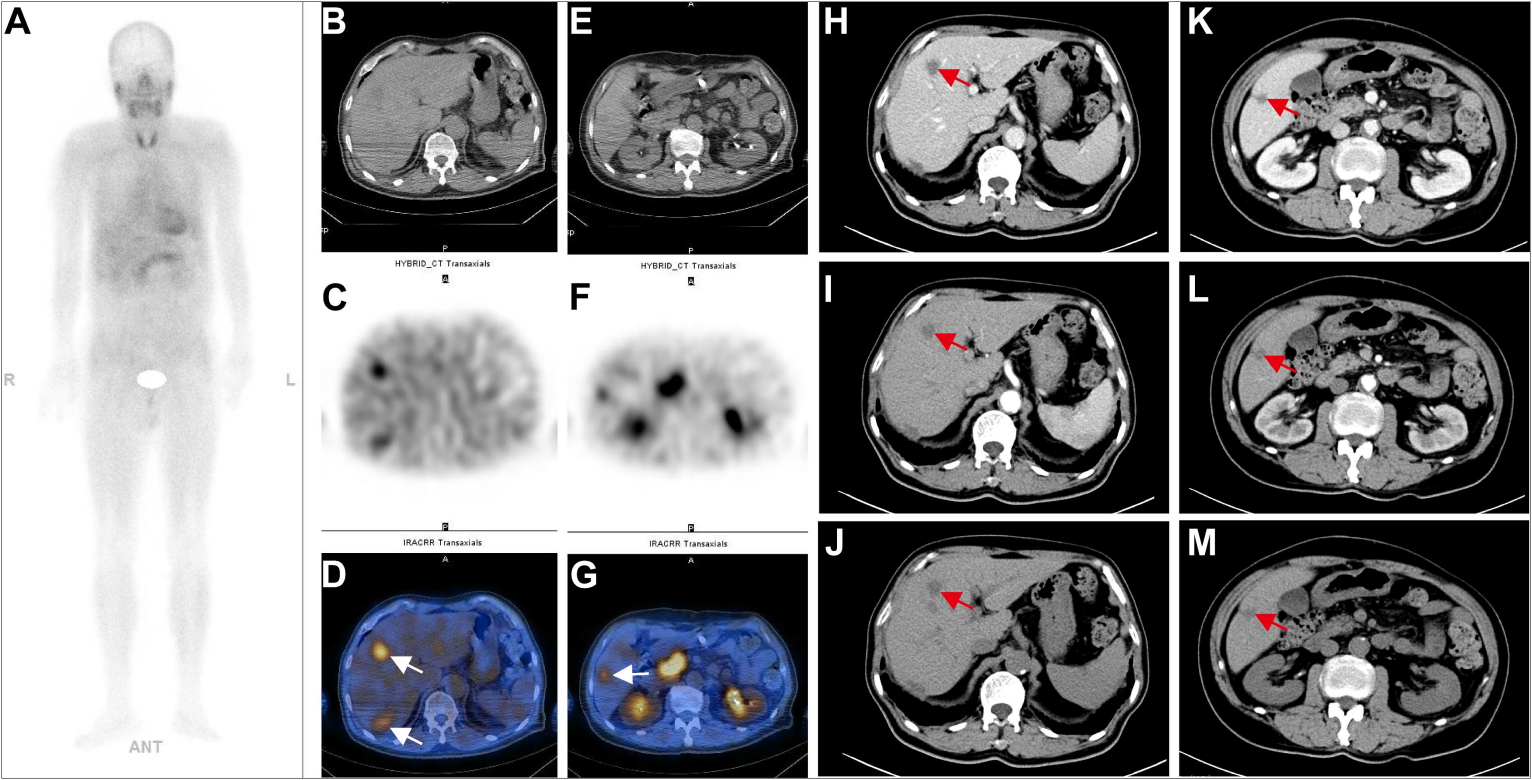
A 73-year-old postoperative male patient with sigmoid colon adenocarcinoma (PT3N1M1, stage II) underwent ^99m^Tc-FAPI-04 SPECT/CT and CECT examinations. The whole-body MIP image **(A)**. The axial CT image **(B,E)**, axial SPECT image **(C,F)**, and the corresponding fused ^99m^Tc-FAPI-04 SPECT/CT image **(D,G)**. In the liver, multiple low-density lesions with unclear margins and high uptake of ^99m^Tc-FAPI-04 were observed on whole-body SPECT/CT. In abdominal CECT, multiple slightly low-density nodular shadows with unclear margins, which exhibited uneven enhancement but clear margins after contrast, can be seen in the left lobe **(H–J)** and the right posterior segment **(K–M)** of the liver, consistent with the imaging manifestations of liver metastases. The white arrow indicates ^99m^Tc-FAPI-04 uptake in the lesion, and the red arrow indicates the metastatic foci in the liver.

**Figure 3:**
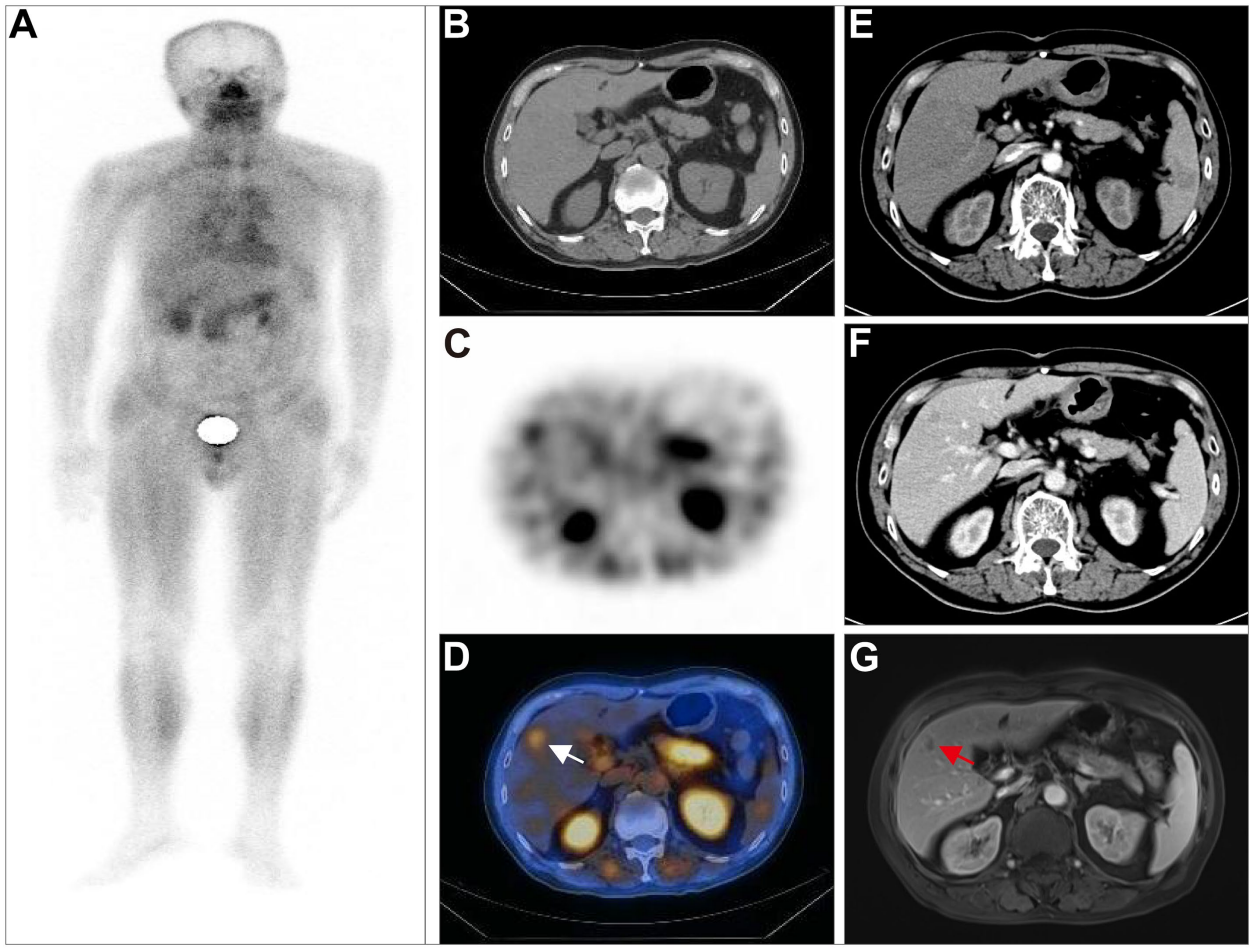
A 75-year-old postoperative male patient with transverse colon adenocarcinoma (pT3N0M0, stage IIA) underwent ^99m^Tc-FAPI-04 SPECT/CT, CECT examination, and MRI for follow-up evaluation. The whole-body MIP image **(A)**. The axial CT image **(B)**, axial SPECT image **(C)**, and fused ^99m^Tc-FAPI-04 SPECT/CT image **(D)**, where radiotracer uptake was observed in the anterior lobe of the liver, with a TBR of 4.26. CECT showed a small patchy low-density shadow in the right anterior lobe of the liver, with a mild enhancement at the edges in the venous phase and delayed phase **(E,F)**. Although the lesion was suspicious, it could not be definitively diagnosed as liver metastases in CECT. The MRI examination **(G)** showed a nodule with low signal intensity on T1-weighted images and high signal intensity on T2-weighted images in the right anterior lobe of the liver. The lesion displayed peripheral ring-like but heterogeneous enhancement after administering the contrast agent, suggesting high chances of liver metastasis. The white arrow indicates high ^99m^Tc-FAPI-04 uptake in liver metastases, and the red arrow indicates the suspected metastatic lesion.

In the patient-based analysis, no metastasis was detected in 4 postoperative CRC patients, as confirmed by the two imaging methods (Figure 1). Among 15 CRC patients, postoperative metastasis was detected in 14 patients and 12 patients through ^99m^Tc-FAPI-04 SPECT/CT and CECT, respectively, suggesting that the two imaging methods were effective for detecting postoperative metastasis of CRC (Figure 2). However, ^99m^Tc-FAPI-04 SPECT/CT had greater sensitivity than CECT (93.3% vs. 80.0%, *χ*^2^ = 4.286, *p* = 0.038). The different types of follow-up imaging examination of a postoperative CRC patient are shown in Figure 3; the atypical imaging features of liver metastases on CECT prevented the radiologists from making a definitive diagnosis, but the ^99m^Tc-FAPI-04 probe was effectively taken up by the metastatic lesions, which immensely helped us evaluate the clinical disease status.

**Figure 4:**
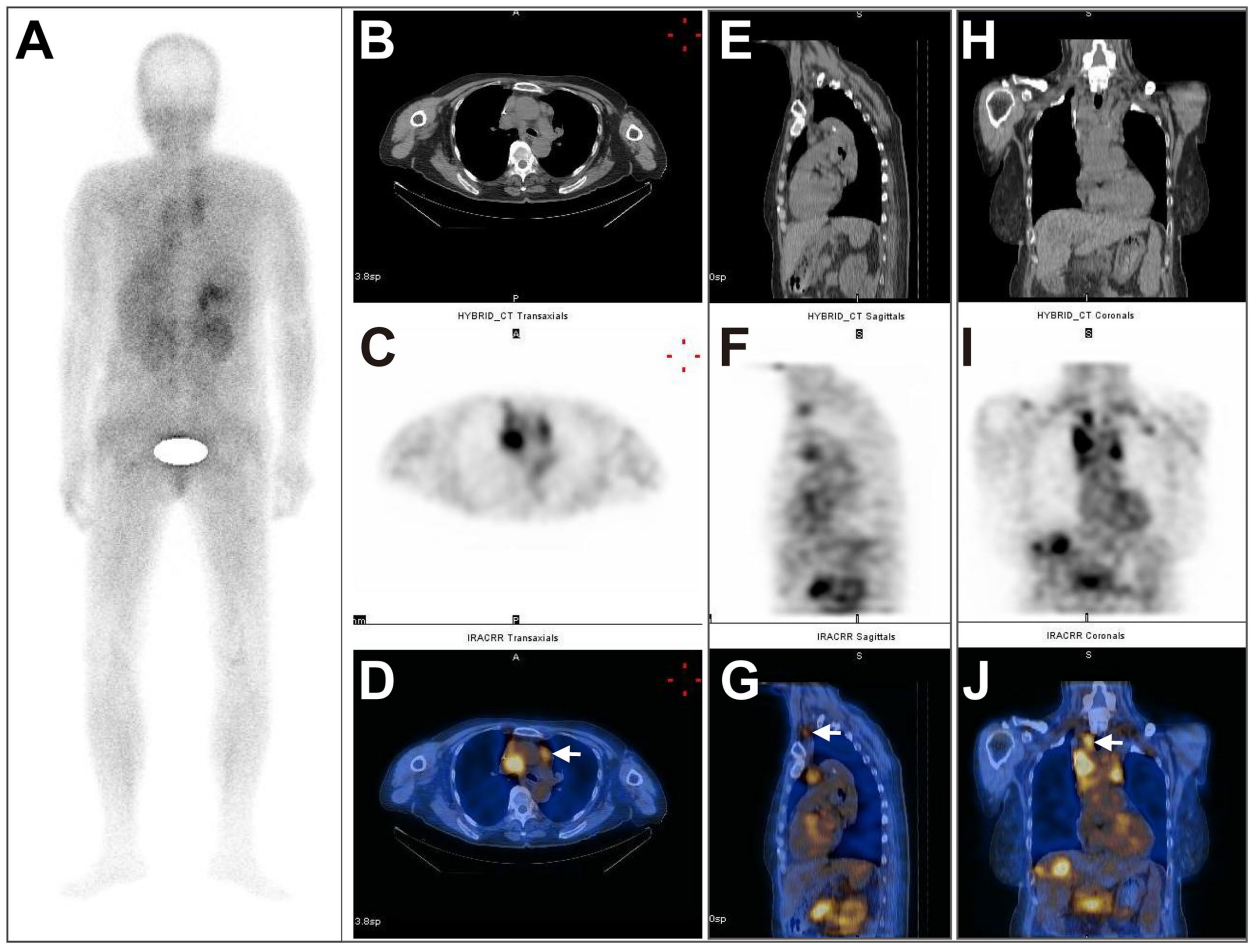
A 66-year-old postoperative female patient with sigmoid colon adenocarcinoma (pT3N0M1, stage II) underwent ^99m^Tc-FAPI-04 SPECT/CT and CECT examinations. The whole-body MIP image **(A)**. The axial **(B)**, coronal **(E)**, and sagittal **(H)** CT images, the axial **(C)**, coronal **(F)**, and sagittal **(I)** SPECT images, and the corresponding fused ^99m^Tc-FAPI-04 SPECT/CT images **(D,G,J)**, where a high accumulation of ^99m^Tc-FAPI-04 occurred not only in the lymph nodes in the neck, supraclavicular fossa, mediastinum, hepatic portal area, and retroperitoneum but also in the liver and right adrenal gland. The white arrow indicates high ^99m^Tc-FAPI-04 uptake in metastatic lesions.

In total, 51 metastatic lesions were found in 15 patients, including 19 liver metastases, 20 lymph node metastases, 7 lung metastases, 1 spleen metastasis, 1 adrenal gland metastasis, and 3 bone metastases (Table 2). The results of ^99m^Tc-FAPI-04 SPECT/CT imaging and CECT examination found 47 lesions and 37 lesions, respectively, with a significant difference between the two methods (92.16% vs. 72.55%, *χ*^2^ = 4.927, *p* = 0.026). Especially, regarding the detection of liver and lymph node metastases, ^99m^Tc-FAPI-04 SPECT/CT imaging showed higher sensitivity than CECT (Table 2). SPECT imaging can be used for total-body scanning, which helps find more metastatic lesions. For example, in one postoperative CRC patient with multiple metastatic lesions, compared to CECT, the ^99m^Tc-FAPI-04 SPECT/CT imaging technique detected additional cervical lymph node metastasis (Figure 4).

**Table 2:** The results of ^99m^Tc-FAPI-04 SPECT/CT and CECT examinations for detecting metastatic lesions (*n* = 15).

Imaging method	The number of lesions						
	Liver (19)	Lymph node (20)	Lung (7)	Spleen (1)	Adrenal gland (1)	Bone (3)	Total (51)
SPECT/CT	18	18	6	1	1	3	47
CECT	15	13	7	0	1	1	37
*χ*2	3.958	4.127	—	—	—	—	4.927
*p*	0.047	0.042	—	—	—	—	0.026

FAPI-04, fibroblast activation protein inhibitor-04; SPECT/CT, single-photon emission computed tomography/X-ray computed tomography; CECT, contrast-enhanced CT.

### 3.3 Comparison of TBR in liver metastasis and lymph node metastasis

According to the ^99m^Tc-FAPI-04 SPECT/CT image, the radioactive counts per unit volume of lymph node metastases were significantly higher than that of liver metastases (*p* = 0.017). Additionally, significantly higher TBR values were found in lymph node metastases than in liver metastases (*p* = 0.010) (Table 3).

**Table 3:** A comparison of the radioactive counts per unit volume and the TBR in liver and lymph node metastases.

Metastasis	The number of lesions	Radioactive counts per unit volume		TBR
		Lesions	Muscle	
Liver	18	911.58 ± 296.59	256.11 ± 77.29	3.61 ± 1.16
Lymph	18	1286.04 ± 546.35	288.79 ± 77.06	4.48 ± 1.61
*p*		0.017	0.213	0.010

TBR, tumor-to-background ratio.

## 4 Discussion

Imaging examination plays an important role in the diagnosis, staging, re-staging, metastatic spread, and treatment monitoring of CRC (14–16). In the clinical setting, CECT is a recommended imaging modality for evaluating postoperative CRC patients with suspected tumor recurrence or distant metastasis (17). However, CECT has several limitations, such as the iodine contrast agent-induced nephropathy in patients with renal impairment, allergic reactions, and particularly, a limited scan range; due to these reasons, it has a good chance of missing some metastatic lesions (18). In contrast, nuclear medicine whole-body functional imaging modality facilitates the evaluation of tumor metabolism, proliferation, activity and parameters affecting treatment outcomes, and has unique advantages in monitoring the post-therapy recurrence and metastasis of tumor. Currently, the radionuclide-labeled FAPI is one of the most attractive novel nuclear medicine molecular probes, and the effectiveness of FAPI-based PET/CT for the diagnosis of various tumors has been evaluated as compared with ^18^F-FDG PET/CT. For example, in the detection of primary and metastatic lung cancer lesions, ^18^F-FAPI PET/CT exhibited higher sensitivity than ^18^F-FDG PET/CT (19); ^68^Ga-FAPI-04 PET/CT was superior to CECT or ^18^F-FDG PET/CT for detecting primary and recurrent CRC, as confirmed by several clinical studies (10–12, 20). Certainly, PET/CT plays a significant role in monitoring the postoperative status of CRC, but this imaging technique bears some drawbacks, including its high cost, relatively complex synthetic method of positron-emitting radiopharmaceuticals. Unlike PET/CT, SPECT/CT is a cheaper and readily available modality. The single-photon emitting radionuclide ^99m^Tc can be got easily from ^99^Mo/^99m^Tc generator, and clinically, ^99m^Tc-labeled radiopharmaceuticals SPECT/CT serves as a simple, economical, convenient and widespread diagnostic procedure. For these reasons, SPECT/CT has received much attention from researchers, particularly with the application of novel molecular probes. For example, ^99m^Tc-PSMA and ^99m^Tc-FAPI SPECT/CT can be used as a substitute for ^68^Ga-PSMA and ^68^Ga-FAPI PET/CT to some extent in the evaluation of specific tumors, respectively (21). Thus, determining the efficacy of ^99m^Tc-FAPI-04 SPECT/CT in the diagnosis of metastasis after radical resection of CRC and assessing its superiority over CECT are important steps.

Among the 19 CRC patients recruited in this study who underwent radical operations, tumor metastasis was found in 15 patients, and the postoperative metastasis rate reached 78.95%. Reliable assessment of the disease status was the prerequisite for making appropriate treatment plans. Among all participants, 4 postoperative CRC patients without metastasis were diagnosed as negative in ^99m^Tc-FAPI-04 SPECT/CT imaging and CECT examination, which indicated that both techniques had high specificity in evaluating metastatic lesions, but SPECT/CT imaging displayed higher sensitivity. For the sake of accurately detecting tumor metastasis, ^99m^Tc-FAPI-04 SPECT/CT is a better choice than CECT for the postoperative follow-up examination of CRC. Overall, the patient-based analysis showed that the ^99m^Tc-FAPI-04 SPECT/CT imaging technique is highly valuable in detecting metastasis in postoperative CRC patients. However, a multicenter clinical trial with a larger sample size is needed to validate this conclusion.

Whole-body scanning and functional imaging techniques, which show the activity of the key molecules of tumors without relying on the anatomical changes in lesions, use ^99m^Tc-FAPI-04 SPECT/CT with a good ability to more accurately find metastatic lesions in earlier stages. Among the 51 lesions detected in 15 postoperative CRC patients, 47 lesions showed high uptake of ^99m^Tc-FAPI-04, mainly in the liver (18 lesions), lymph nodes (18 lesions), and lungs (6 lesions). However, no radiotracer uptake was found in a few metastatic lesions in lymph nodes (2 lesions), liver (1 lesion), and lungs (1 lesion). One reason for this false-negative result is that these metastatic lesions were too small, with a maximum diameter of <0.5 cm. In CRC tumor tissue, FAP is mainly expressed by CAFs, and the amount and activity of CAFs largely determine the expression of FAP. Therefore, tiny metastatic lesions with a small quantity of CAFs have negligible uptake of ^99m^Tc-FAPI-04. Also, a high concentration of ^99m^Tc-FAPI-04 was detected in the abdominal surgical scar and lung fibrosis tissue in 2 postoperative CRC patients, which occurred because fibroblasts in these tissues were activated along with the overexpression of FAP (22–24). To reduce the rate of misdiagnosis, a detailed analysis of history, clinical symptoms, and relevant physical examination is needed to interpret the ^99m^Tc-FAPI-04 SPECT/CT results.

Although CECT is used as the first-line diagnostic tool for detecting recurrent and metastatic CRC, it has some limitations when used alone. The efficacy of CECT lies in its ability to provide the anatomical information of lesions, but the tiny lesions and the distant metastatic lesions may be missed. By comparison, SPECT/CT provides physiological information along with anatomical information, and may be a better alternative to assess the disease progression of the patients undergoing therapy. For example, ^99m^Tc-Sestamibi SPECT/CT has been reported to have an incremental value over CECT for differentiating malignant or aggressive renal tumors from benign or indolent ones, because the former can give the pathophysiological characteristics of different types of diseases (25). According to our present study, among the 51 postoperative metastatic lesions mentioned above, only 37 lesions could be diagnosed clearly by CECT, including 15 liver metastases and 13 lymph node metastases; in both types of metastases, its diagnostic performance was inferior to that of ^99m^Tc-FAPI-04 SPECT/CT. The remaining 13 lesions were undetectable, indeterminate, or negative. Thus, CECT missed 4 liver metastatic lesions in 3 patients, including 2 patients with 2 lesion and 1 patient with 2 lesions. In CECT images, these lesions generally had a small low-density area with weak enhancement, without the typical features of CRC liver metastases. The lesion volume, tube current, and slice thickness influenced the image quality of CECT, resulting in its low diagnostic efficacy in liver metastasis (26, 27). Our results were similar to those of another study, in which it was reported that ^99m^Tc-FAPI SPECT/CT has greater diagnostic efficiency than CECT in detecting the distant metastasis of patients with digestive system tumors, especially in identifying liver metastases (28). Additionally, 7 cervical and mediastinal lymph node metastases, 1 spleen metastasis, and 2 bone metastases were missed by CECT, indicating that the ability of CECT to detect distant metastases in postoperative CRC patients is limited. Namely, SPECT/CT is a more sensitive and accurate tool for evaluating distant metastases than CECT.

With the development of iterative image reconstruction by incorporating compensations for collimator-detector response, attenuation and scatter, SPECT quantification in terms of standard uptake value (SUV) or kBq/cc become feasible, providing a more accurate method for monitoring tumor metabolic response. Regretfully, our SPECT/CT system did not incorporate the tools or algorithms for generating quantitative SPECT data, and we chose TBR as an alternative tool to assess the uptake of ^99m^Tc-FAPI-04 in metastases. In this study, lymph node metastases had a higher and more stable uptake of ^99m^Tc-FAPI-04 than liver metastases, along with a higher TBR value. By analyzing the SPECT/CT image of liver metastases, we found that liver lesions varied in size from less than 1 cm to over 8 cm, and the huge metastatic lesions had several small areas of necrotic tissues with low density, leading to the non-uniform uptake of ^99m^Tc-FAPI-04 by the lesions. In contrast, lymph node metastases were relatively small, with a maximum diameter of ≤3 cm, homogeneous density, and uniform distribution of radiotracer. The combination of the above factors facilitated a more significant accumulation of ^99m^Tc-FAPI-04 and a higher TBR in metastatic lymph nodes, suggesting that ^99m^Tc-FAPI-04 SPECT/CT is a highly promising tool for the radiological follow-up of postoperative CRC at risk of lymph node metastasis.

## 5 Conclusion

To summarize, compared to CECT, ^99m^Tc-FAPI-04 SPECT/CT showed higher sensitivity in detecting metastatic lesions, especially liver and lymph node metastases. These findings indicated that it may be used to assess metastases in postoperative patients with CRC in routine clinical practice.

## Data Availability

The original contributions presented in the study are included in the article/Supplementary material, further inquiries can be directed to the corresponding authors.
