# Supplementary material for: 99mTc-FAPI-04 SPECT/CT outperforms contrast-enhanced CT in detecting metastasis in postoperative patients with colorectal cancer
Source: Front Med (Lausanne). 2024 Sep 23;11:1462870. doi: 10.3389/fmed.2024.1462870 (PMC11456442; doi:10.3389/fmed.2024.1462870)
Supplement: Supplementary file 1 [file Data_Sheet_1.PDF]

Supplementary Material

^{99m}Tc-FAPI-04 SPECT/CT Outperforms Contrast-Enhanced CT in Detecting Metastasis in Postoperative Patients with Colorectal Cancer

Donghua Sun[1,#], Li Ma[2,#], Yan Liu[1,#], Caili Bao[1], Guorong Jia[2], Tao Wang[2,*], Yingqiu Wang[1,*]

[1]Department of Nuclear Medicine, Yangpu Hospital, School of Medicine, Tongji University, Shanghai, People's Republic of China.

[2]Department of Nuclear Medicine, the First Affiliated Hospital of Naval Medical University, Shanghai, People's Republic of China.

#These authors contributed equally to this work.

*** Correspondence:**
Corresponding Authors. Tao Wang, E-mail: wangtao2086@smmu.edu.cn; Yingqiu Wang, E-mail: wangyingqiu2023@outlook.com.

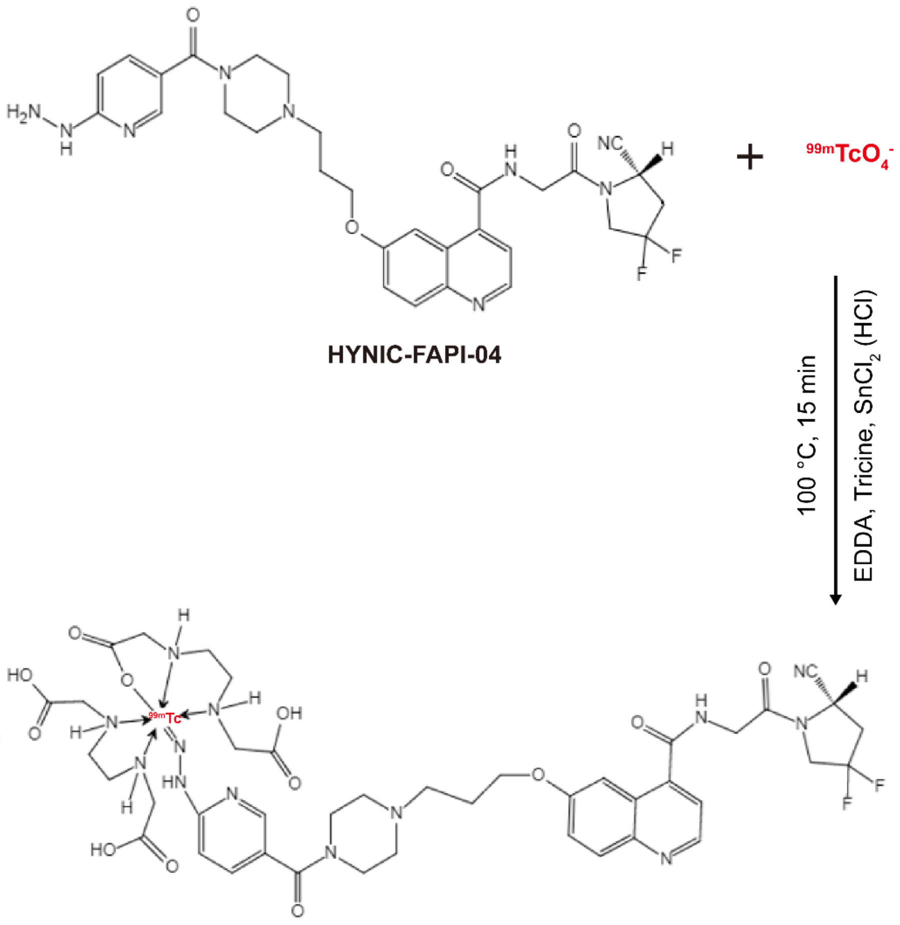

99mTc-HYNIC-FAPI-04
(abbreviated as 99mTc-FAPI-04)

Figure S1. The synthetic process of ^{99m}Tc-FAPI-04.

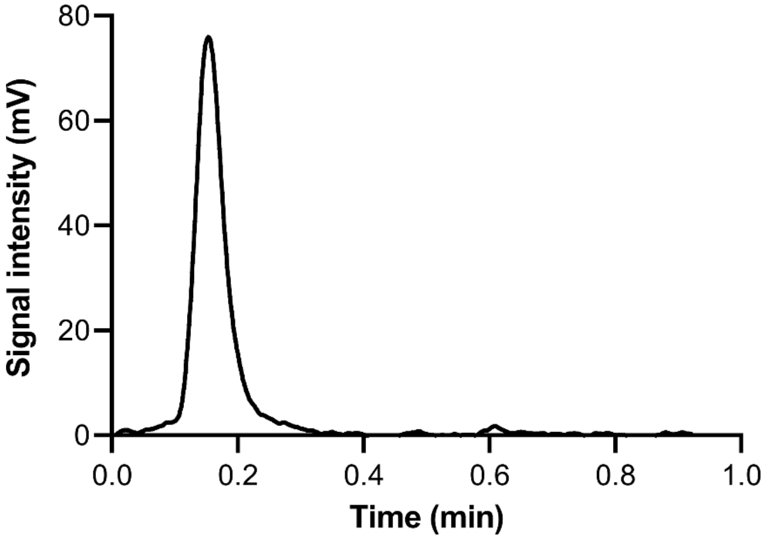

Figure S2. The labeling rate of ^{99m}Tc-FAPI-04 is usually over 95%.
